# A phase II study of sequential 5-fluorouracil, epirubicin and cyclophosphamide (FEC) and paclitaxel in advanced breast cancer (Protocol PV BC 97/01)

**DOI:** 10.1054/bjoc.2001.1897

**Published:** 2001-07

**Authors:** A Riccardi, P Pugliese, M Danova, S Brugnatelli, D Grasso, M Giordano, G Bernardo, G Giardina, S Fava, G Montanari, C Pedrotti, G Trotti, E Rinaldi, M A Poli, C Tinelli

**Affiliations:** Medicina Interna ed Oncologia Medica, Servizio di Biometria ed Epidemiologia Clinica, Università and IRCCS Policlinico S. Matteo, Pavia, 27100; Servizio di Oncologia, Ospedale S. Anna, Como, 22100; Servizio di Oncologia Medica e Riabilitazione, Fondazione Maugeri, Pavia, 27100; Centro Senologico, Ospedale di Circolo, Varese, 21100; Divisione di Medicina II, Ospedale di Legnano, Legnano, 20025; Divisione di Medicina Generale, Ospedale di Lodi, Lodi, 20075; Divisione di Medicina, Ospedale di Sondalo, Sondalo, 23035; Divisione di Medicina Generale II, Ospedale di Busto Arsizio, Busto Arsizio, 21052; Divisione di Medicina I, Ospedale di Magenta, Magenta, 20013; Divisione di Radioterapia, Ospedale di Cremona, Cremona, 26100, Italy

**Keywords:** advanced breast cancer, anthracycline-containing regimen, paclitaxel, sequential chemotherapy

## Abstract

Sequential administration of the association of 5-fluorouracil, epirubicin and cyclophosphamide (FEC) and paclitaxel could be better tolerated than the association of an anthracycline and paclitaxel while having a similar antitumour effect. 69 patients with advanced breast cancer previously untreated with anthracyclines or paclitaxel entered a phase II multicentre study in which FEC was followed by paclitaxel. Both regimens were administered 4 times every 21 days. The median follow-up is 20 months and 38/69 patients have died. Grade III–IV toxicity was acceptable. Leukopenia occurred in 26% of patients, thrombocytopenia in 2% and anaemia in 4%. One patient had reversible heart failure during FEC therapy. Peripheral neuropathy and arthralgia-myalgia occurred in 9% and 4% of patients, respectively and one patient had respiratory hypersensitivity during paclitaxel treatment. 9 patients did not complete therapy because of: treatment refusal (*n*= 1), cardiac toxicity (*n*= 1), early death during FEC chemotherapy (*n*= 1), major protocol violations (*n*= 4), hypersensitivity reaction (*n*= 1) and early death during paclitaxel chemotherapy (*n*= 1). The overall response rate was 65% (95% CI = 53–76), and 7% of patients had stable disease. Therapy was defined as having failed in 28% of patients because they were not evaluable (13%) or had progressive disease (15%). The median time to progression and survival are 13.2 and 23.5 months, respectively. Sequential FEC-paclitaxel is a suitable strategy for patients with metastatic breast cancer who have not been previously treated with anthracyclines and/or taxanes. In fact, it avoids major haematologic toxicity and has a good antitumour effect. © 2001 Cancer Research Campaign http://www.bjcancer.com


*British Journal of Cancer* (2001) **85**(2), 141–146
© 2001 Cancer Research Campaign
doi: 10.1054/ bjoc.2001.1897, available online at http://www.idealibrary.com on IDEAL®

http://www.bjcancer.com

### A phase II study of sequential 5-fluorouracil, epirubicin and cyclophosphamide (FEC) and paclitaxel in advanced breast cancer (Protocol PV BC 97/01)

**A Riccardi[1], P Pugliese[1], M Danova[1], S Brugnatelli[1], D Grasso[1], M Giordano[2], G Bernardo[3], G Giardina[4], S Fava[5], G Montanari[6], C Pedrotti[7], G Trotti[8], E Rinaldi[9], MA Poli[10] and C Tinelli[11]**

[1]Medicina Interna ed Oncologia Medica and [11]Servizio di Biometria ed Epidemiologia Clinica, Università and IRCCS Policlinico S. Matteo, 27100 Pavia; [2]Servizio di Oncologia, Ospedale S. Anna, 22100 Como; [3]Servizio di Oncologia Medica e Riabilitazione, Fondazione Maugeri, 27100 Pavia; [4]Centro Senologico, Ospedale di Circolo, 21100 Varese; [5]Divisione di Medicina II, Ospedale di Legnano, 20025 Legnano; [6]Divisione di Medicina Generale, Ospedale di Lodi, 20075 Lodi; [7]Divisione di Medicina, Ospedale di Sondalo, 23035 Sondalo; [8]Divisione di Medicina Generale II, Ospedale di Busto Arsizio, 21052 Busto Arsizio; [9]Divisione di Medicina I, Ospedale di Magenta, 20013 Magenta; [10]Divisione di Radioterapia, Ospedale di Cremona, 26100 Cremona, Italy

**Summary** Sequential administration of the association of 5-fluorouracil, epirubicin and cyclophosphamide (FEC) and paclitaxel could be better tolerated than the association of an anthracycline and paclitaxel while having a similar antitumour effect. 69 patients with advanced breast cancer previously untreated with anthracyclines or paclitaxel entered a phase II multicentre study in which FEC was followed by paclitaxel. Both regimens were administered 4 times every 21 days. The median follow-up is 20 months and 38/69 patients have died. Grade III–IV toxicity was acceptable. Leukopenia occurred in 26% of patients, thrombocytopenia in 2% and anaemia in 4%. One patient had reversible heart failure during FEC therapy. Peripheral neuropathy and arthralgia-myalgia occurred in 9% and 4% of patients, respectively and one patient had respiratory hypersensitivity during paclitaxel treatment. 9 patients did not complete therapy because of: treatment refusal ($n$ = 1), cardiac toxicity ($n$ = 1), early death during FEC chemotherapy ($n$ = 1), major protocol violations ($n$ = 4), hypersensitivity reaction ($n$ = 1) and early death during paclitaxel chemotherapy ($n$ = 1). The overall response rate was 65% (95% CI = 53–76), and 7% of patients had stable disease. Therapy was defined as having failed in 28% of patients because they were not evaluable (13%) or had progressive disease (15%). The median time to progression and survival are 13.2 and 23.5 months, respectively. Sequential FEC-paclitaxel is a suitable strategy for patients with metastatic breast cancer who have not been previously treated with anthracyclines and/or taxanes. In fact, it avoids major haematologic toxicity and has a good antitumour effect. © 2001 Cancer Research Campaign http://www.bjcancer.com

**Keywords**: advanced breast cancer; anthracycline-containing regimen; paclitaxel; sequential chemotherapy

Although metastatic breast cancer (MBC) is considered a chemosensitive tumour, its palliative treatment remains a challenge. Drug associations are usually employed as first line treatment, despite the controversy (Winer et al, 2001) as to whether combination chemotherapy does or does not provide greater antitumour effect than single agents used at appropriate dose levels (Sledge et al, 1997; Bishop et al, 1999; Nabholtz et al, 1999).

Anthracycline-containing regimens, administered for 6–8 courses, are probably the first choice therapy in patients who have not received an anthracycline as adjuvant therapy. The association of doxorubicin or epirubicin with 5-fluorouracil and cyclophosphamide (FAC or FEC regimens) produces a higher response rate (RR) and longer time to progression than CMF-like regimens (Fossati et al, 1998), and a survival advantage is confirmed in the adjuvant setting (Coombes et al, 1996; Levine et al, 1998). From several trials on MBC, anthracycline-containing regimens produce a RR of about 65% (with a 16% rate of complete responses), a progression-free survival of 11.5 months and an overall survival of 21 months (Rahman et al, 1999).

*Received 18 January 2001*
*Revised 17 April 2001*
*Accepted 2 May 2001*

*Correspondence to:* A Riccardi

Ways of ameliorating the treatment of MBC have included increasing the drug dose-intensity, maintenance therapy and use of new drugs.

No clear advantage has been obtained from doubling the anthracycline dose intensity within the FEC regimen (Biganzoli and Piccart, 1997; Riccardi et al, 2000).

The relevance of continuing long-lasting combination chemotherapy following FEC, as a maintenance, is also largely unsettled. This approach has increased the time to progression, but the survival advantage was not significant and toxicity was increased (Muss et al, 1991; Falkson et al, 1998).

New treatment opportunities to be explored come from the availability of new drugs that are both highly effective and not cross-resistant with the anthracyclines. Taxanes have substantial activity in previously treated patients (Holmes et al, 1991; Reichman et al, 1993), including a 30–50% RR in anthracycline-resistant disease (Gehl et al, 1996; Nabholtz et al, 1996; Pivot et al, 1999; Rivera et al, 2000).

Combined with doxorubicin, paclitaxel produces a higher RR than either paclitaxel or doxorubicin alone (Sledge et al, 1997; Pouillart et al, 1999), but myelosuppressive and mucosal toxicities are substantially increased. This happens particularly when paclitaxel is administered shortly before the anthracycline, because paclitaxel lowers doxorubicin elimination, especially with long-lasting infusions (Conte et al, 1997; Venturini et al, 2000).

Another way of treatment could be sequencing FEC with taxanes as first-line therapy. Antitumour advantages from FEC could be enhanced by the addition of a sequential non-cross-resistant drug for a short period. Avoiding combination chemotherapy is also expected to lower overall toxicity.

We used an integrated treatment with sequential FEC and paclitaxel in a multicenter phase II study as first or second line treatment in MBC patients previously untreated with anthracyclines or taxanes as adjuvant therapy (Protocol PV BC 97/01).

### PATIENTS AND METHODS

Between January, 1998, and July, 1999, a phase II multicentre study (PV BC 97/01) enrolled 69 consecutive patients with MBC who had not previously received anthracyclines or taxanes as adjuvant therapy or as first-line therapy for metastatic disease. Patients were treated with sequentially administered FEC and paclitaxel therapy.

The study was approved by the Clinical Research Review Board of the Department of Internal Medicine of University of Pavia and IRCCS Policlinico S. Matteo, and written informed consent was obtained from each patient.

### Eligibility and exclusion criteria

Patients had progressive MBC and had not previously received anthracyclines or taxanes.

Eligibility criteria were: histologic or cytologic proof of primary breast cancer; presence of at least one metastatic lesion bidimensionally measurable by physical examination and/or radiologic means; age between 18 and 70 years; performance status (PS) ≤ 2 (World Health Organization, WHO, scale); life expectancy > 6 months; normal blood counts and biochemistry (absolute granulocyte, WBC, count > $2.0 \times 10^9$ $l^{-1}$, platelets, PLT, count > $100 \times 10^9$ $l^{-1}$, bilirubin < 34 μmol $l^{-1}$, creatinine < 106 μmol $l^{-1}$) and normal cardiac function (i.e., normal ECG and 2-dimensional echocardiography showing a left ventricular ejection fraction, LVEF, > 50%).

Exclusion criteria included pregnancy or lactation, childbearing potential without adequate contraception, preexisting grade II or higher motosensorial neurotoxicity, and concomitant treatment with other experimental drugs.

Drop-out criteria were WHO grade IV non-haematologic toxicity, symptomatic heart failure or LVEF reduction to < 50% of pretreatment value, refusal to continue participation in the study, loss to follow-up, or major treatment violations. All these patients were considered as non-responders, so that results are described on an intention-to-treat basis.

### Treatment

Patients received 4 courses of FEC (mg $m^{-2}$: 5-fluorouracil 600, epirubicin 60, cyclophosphamide 600) chemotherapy followed by 4 courses of paclitaxel (175 mg $m^{-2}$ as a 3 h i.v. infusion) chemotherapy. Before paclitaxel, patients were premedicated with prednisone (125 mg p.o. 12 and 6 h before paclitaxel), ranitidine (300 mg p.o. 12 h and 300 mg i.v. 1 h before paclitaxel) and diphenhydramine (10 mg i.v. just before paclitaxel). Both FEC and paclitaxel were administered every 21 days.

Ondansentron or granisetron were used as antiemetics. Patients with leukopenia (WBC ≤ $1.0 \times 10^9$ $l^{-1}$) received oral ciprofloxacin (500 mg twice a day) as antibiotic prophylaxis. If the patient developed fever > 38.0˚C and neutropenia, they were hospitalized and treated with i.v. netilmicin (2 mg $kg^{-1}$) and piperacillin (2 g) twice a day. If fever persisted beyond 6 days despite antibiotic treatment, i.v. fluconazol (200 mg twice a day) was added. No prophylactic use of haematopoietic growth factors was planned. However, if granulocytes were < $0.5 \times 10^9$ $l^{-1}$ on day 14, the patients were given subcutaneous granulocyte colony-stimulating factor (G-CSF, 5 μg $kg^{-1}$ $day^{-1}$). Clodronate (600–900 mg $week^{-1}$) or pamidronate (90 mg/3 weeks) were given by i.v. infusion to patients with bone lesions.

Toxic effects of treatments were assessed according to WHO criteria.

### Treatment monitoring

Pre-treatment evaluation included a complete medical history, clinical examination, complete blood counts and biochemistry, ECG, chest X-ray, liver ultrasounds, bone scan and, if indicated, computed tomography. Bone marrow aspiration and/or biopsy were performed when blood cell counts were unexplainably abnormal.

Before each FEC or paclitaxel course, physical examination was performed. Blood counts were obtained on days 10, 14 and 21 of each course. Before starting the subsequent drug administration (i.e. on day 21 of each chemotherapy course), guidelines were given for delaying treatment or reducing its dosage. If platelets were < $100 \times 10^9$ $l^{-1}$ and/or WBC < $3.0\times 10^9$ $l^{-1}$, a week's delay in resuming chemotherapy had to be observed. If the PLT or WBC count was still low after this delay, all drug dosages were halved.

Following the fourth course of FEC and following the fourth course of paclitaxel, the whole pre-treatment evaluation was repeated to assess response to therapy.

### Criteria for tumour response and toxicity

Tumour responses and toxic effects of treatment were assessed, using WHO criteria, after 4 courses of FEC and again after 4 courses of paclitaxel. For those patients with only assessable bone metastases, the UICC criteria for skeletal disease were used. As defined by these criteria, partial or complete responses require recalcification of lytic lesions while disease progression is enlargement of an already existing lesions or the appearance of new lesions.

The choice of evaluating response following the fourth course of FEC was based on our previous data which indicated that response to FEC was similar following 3 and 6 courses of this regimen (Riccardi et al, 2000).

Duration of response was taken to be the period from the end of successful induction therapy until relapse, and surviving patients who had not relapsed during the follow-up were censored from the data analysis. Patients who died before relapse were considered as events.

Time to progression (TTP) was defined as the time from starting treatment to when progression of the disease was first documented.

Survival was the time from starting treatment to death.

 © 2001 Cancer Research Campaign

### Statistical analysis

This phase II, non-randomized, open-labelled study, required about 70 patients to be enrolled, as calculated by Gehan's method accounting for the principal end-point, i.e. response to treatment, in order for results to be statistically meaningful. The expected complete or partial RR was 60%, with 5% false positives and a power of 90%. The planned study duration was 24 months, providing a median 12 month-follow-up period after treatment discontinuation.

The Kaplan–Meier methodology was used for plotting response duration, TTP and overall survival.

### RESULTS

The main clinical characteristics of the 69 recruited patients are reported in Table 1.

Median age was 54 (range: 34–70) years. Of the 69 patients, 27 had received adjuvant hormone therapy and 30 adjuvant CMF chemotherapy. 59 patients had single or multiple visceral involvement. 2 patients had bone disease only.

A total of 488 chemotherapy courses (88% of those planned) were administered, namely 264 of FEC (96% of those planned) and 224 of paclitaxel (81% of those planned). The mean number of delivered courses was 3.8/patient for FEC and 3.3/patient for paclitaxel treatment. A number of courses were not administered because 9 patients did not complete therapy, for the reasons reported below.

At time of this analysis (July 2000), the median follow-up of all recruited patients is 20 (range: 2–32) months and 38 of 69 (55%) patients have died.

**Table 1** Characteristics of patients with metastatic breast cancer who were treated with first-line sequential 5-fluorouracil + epirubicin + cyclophosphamide (FEC) and paclitaxel therapy

| | |
|---|---|
| No. of entered patients | 69 |
| Age, years | |
| Median | 54 |
| Range | 34–70 |
| WHO performance status | |
| 0 | 37 |
| 1 | 22 |
| 2 | 10 |
| DFI, months | |
| Median | 36 |
| Range | 0–216 |
| Prior radiotherapy | 10 |
| Prior hormotherapy | 27 |
| Prior adiuvant chemotherapy (CMF) | 30 |
| Sites of disease | |
| Visceral organs ± bone ± soft tissue | 59 |
| visceral organs ≤ 2 | 30 |
| visceral organs > 2 | 29 |
| Bone ± soft tissue | 6 |
| Soft tissue ± locoregional | 4 |
| No of metastatic sites | |
| 1 | 37 |
| 2 | 19 |
| ≥3 | 13 |
| Hormone receptor status | |
| positive | 33 |
| negative | 19 |
| unknown | 17 |

DFI, disease-free-interval; WHO, World Health Organization; CMF: cyclophosphamide, methotrexate and 5-fluorouracil; TAM = tamoxifen.

### Toxicity

Grade III–IV haematologic and non-haematologic toxicities during FEC and paclitaxel treatments, as evaluable in 60 of 69 patients, are detailed in Table 2. Both were acceptable.

As expected, epirubicin was more toxic than paclitaxel in terms of haematologic, gastrointestinal, and cardiac side effects, while paclitaxel induced more neurotoxicity (mainly sensory) and arthralgia/myalgia.

Over the whole sequential treatment, leukopenia occurred in 26% of patients (with no febrile episodes), thrombocytopenia in 2% and anaemia in 4%. Of the courses of FEC and paclitaxel administered, 93% of the former and 91% of the latter were delivered without the need for dose reductions or delay, and did not require the use of G-CSF.

Alopecia was almost universal. Mucositis (stomatitis and/or diarrhea) occurred in 2% of patients. During FEC therapy, symptomatic cardiotoxicity occurred in one patient, who developed reversible heart failure, and LVEF decreased by > 20% in 2 other patients. During paclitaxel treatment, peripheral neuropathy (tingling, numbness and paraesthesia) occurred in 9% of patients and arthralgia and/or myalgia in 4%. One patient had a respiratory hypersensitivity reaction during paclitaxel therapy.

**Table 2** Grade III–IV WHO toxicity in 69 patients with metastatic breast cancer who where treated with first line sequential 5-fluorouracil, epirubicin and cyclophosphamide (FEC) and paclitaxel therapy

| Toxicity | During FEC % of pts | During paclitaxel % of pts | Overall % of pts |
|---|---|---|---|
| Leukopenia | 23 | 14 | 26 |
| Thrombocytopenia | 2 | 1 | 2 |
| Anaemia | 3 | 2 | 4 |
| Nausea and vomiting | 6 | 2 | 7 |
| Alopecia | 88 | 92 | 92 |
| Mucosytis | 2 | 1 | 2 |
| Cardiac toxicity | 1 | 0 | 1 |
| Neuropathy | 0 | 9 | 9 |
| Flu-like symptoms–hypersensitivity | 0 | 1 | 1 |
| Arthralgia–myalgia | 0 | 4 | 4 |

*© 2001 Cancer Research Campaign*

**Table 3** Response rates after 5-fluorouracil, epirubicin, cyclophosphamide (FEC) and after sequential FEC and paclitaxel treatment in 69 patients with metastatic breast cancer

| Response no. of patients % of patients (95% Cl) | After 4 FEC courses | After 4 FEC + 4 paclitaxel courses |
|---|---|---|
| Complete response | 11 | 11 |
| | 16 | 16 |
| | 8–27 | 8–27 |
| Partial response | 34 | 34 |
| | 49 | 49 |
| | 37–62 | 37–62 |
| Stable disease | 17 | 5 |
| | 25 | 7 |
| | 15–36 | 2–16 |
| Progressive disease | 4 | 10 |
| | 6 | 15 |
| | 2–14 | 7–25 |
| Not evaluable | 3 | 9 |
| | 4 | 13 |
| | 2–11 | 7–22 |

Cl = confidence interval.

### Response

Response and stable disease rates following FEC alone and following FEC and paclitaxel are detailed in Table 3.

9 patients did not complete therapy because of treatment refusal (1 patient), reversible heart failure (1 patient), early death that occurred during FEC chemotherapy (1 patient), major protocol violations (4 patients), respiratory hypersensivity reaction (1 patient) and sudden death due to cerebrovascular insufficiency during paclitaxel chemotherapy (1 patient). These 9 patients are evaluated as treatment failures.

At the end of the whole FEC-paclitaxel sequence, overall RR was 65% (95% Cl = 53–76), and 7% of patients had stable disease. Therapy was considered to have failed in 28% (95% Cl = 17–40) of patients either because they could not be evaluated (9 patients, 13%) or because they had progressive disease (10 patients, 15%).

Responses were observed at all sites of measurable disease, except bone, independently of the number of sites involved (≤ 2 or > 2). Both patients with isolated bone disease had stable disease following the whole FEC-paclitaxel treatment.

Of the 11 patients who achieved a CR after FEC, 9 maintained this condition after paclitaxel and 2 became not evaluable after paclitaxel. Of the 34 patients who had a PR after FEC, the status remained unchanged in 28, whereas 2 achieved a CR, 2 progressed and 2 became not evaluable after paclitaxel. Of the 17 patients who had stable disease after FEC, 5 maintained this condition, 6 had a PR, 4 progressed and 2 became not evaluable after paclitaxel. The 4 patients who progressed during FEC therapy also failed to respond to paclitaxel.

### Duration of response and of survival

Median duration of response was 10.1 (2–33) months, median TTP was 13.2 (range 1–32) months and median survival was 23.5 (range: 2–32) months (Figure 1).

### DISCUSSION

In patients with MBC previously untreated with anthracycline or taxanes, the integrated sequential FEC-paclitaxel treatment administered as first or second line therapy produced the same or better antitumour effect than regimens that associate an anthracycline and paclitaxel, with less side effects.

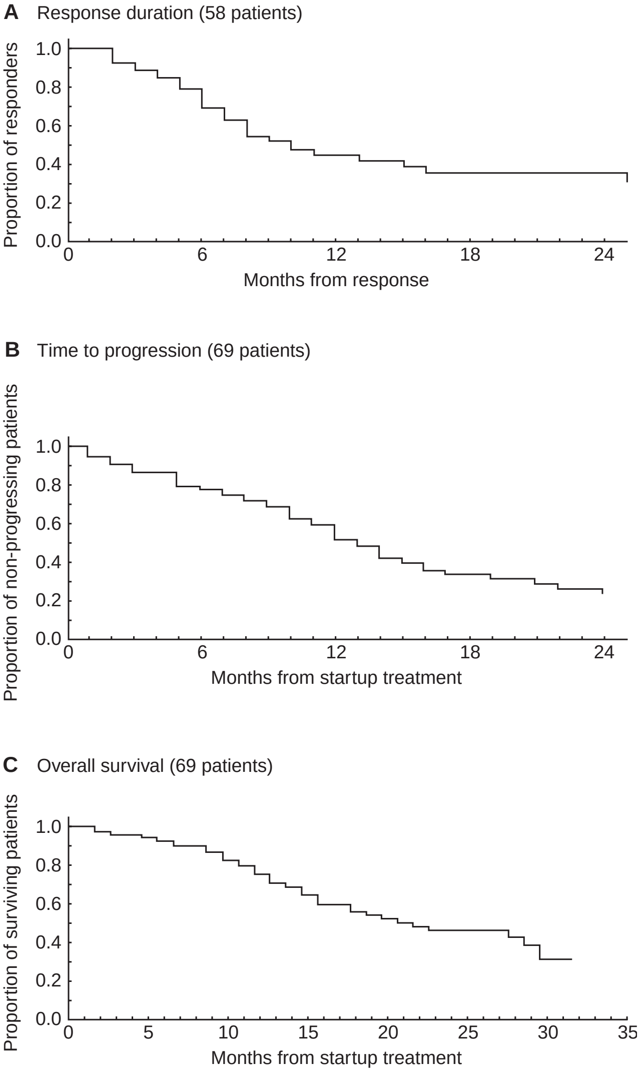

**Figure 1** Duration of response, of time to progression and of survival in patients with metastatic breast cancer who were treated with sequential FEC and paclitaxel

In fact, the overall antitumour effect was similar in our study and in 3 recently published phase II studies (Pazos et al, 1999; Sparano et al, 1999; Rischin et al, 2000) in which an anthracycline was associated with paclitaxel. In these studies, the drug dosages (i.e., epirubicin 75 mg $m^{-2}$ or doxorubicin 50–60 mg $m^{-2}$ and paclitaxel 175–200 mg $m^{-2}$) were comparable to those used in our sequential regimen and most (about 75%) patients were untreated for metastatic disease. The 65% overall RR (with 16% of CR) obtained with sequential FEC-paclitaxel compares well with the overall RR of 52–76% (with 8–14% of CR) reported in these studies. Median duration of response, TTP and survival (10.1, 13.2 and 23.5 months, respectively) in our study were also similar to those in the above-mentioned studies (6.4–13.4, 6.9–7.3, 17.9–21.6 months, respectively). It should be noted that in one of these trials (Sparano et al, 1999) efficacy was not measured on an intention-to-treat basis. Our study and these 3 quoted studies failed to confirm the much higher RR (94%, with 41% complete

*© 2001 Cancer Research Campaign*

response) reported in a previous study (Gianni et al, 1995), that, otherwise, attained a similar duration of response, TTP and survival.

Overall toxic effects of treatments are not easy to compare between studies for several reasons, including how the study was dedicated at eliciting them, how many patients were evaluable for side effects, and the different ways of reporting them (for example, as a percentage of patients or of courses).

Notwithstanding these drawbacks, overall grade III–IV haematologic toxicity was reduced by sequencing FEC and paclitaxel rather than administering an association of an anthracycline and paclitaxel. In fact, leukopenia occurred in 26% of our patients (with no febrile episodes) but in the 55–97% of the patients in previous studies (with 0–14% of febrile episodes). Thrombocytopenia and anaemia occurred in 2 and 4% of patients, respectively, in our study and in 1–29% and 5–21% of patients, respectively, in the quoted studies. A 12% rate of grade III–IV leukopenia has been reported with the association of anthracycline and paclitaxel in an adjuvant setting (Venturini et al, 2000).

Comparing the incidence of non-haematologic side effects among different studies is even harder because of the additional problem of a notable degree of subjectivity entering the evaluation. In our study, symptomatic heart failure occurred in one patient and a > 20% decline in LVEF occurred in 2 other (3%) patients during FEC therapy. Cumulatively, these events occurred in, respectively, 2 and 10% of patients in the anthracycline-paclitaxel association studies. Mucositis, arthralgia-myalgia and neuropathy occurred in 2, 4 and 9%, respectively, of our patients.

A puzzling question is comparing sequential FEC-paclitaxel with regimens in which an anthracycline-containing induction regimen has been randomly followed by a CMF-like regimen as maintenance therapy for about 2 years. These studies (Muss et al, 1991; Falkson et al, 1998) were not analysed on an intention-to-treat basis and randomized only patients who had a complete response (Muss et al, 1991) or either response or stable disease (Falkson et al, 1998). With respect to the outcomes in the control group, maintenance therapy afforded a significantly longer TTP, but overall survival was not increased and the associated grade III–IV haematologic WHO toxicity was substantial. In fact, 3–8% and 3% of patients experienced leukopenia and thrombocytopenia, respectively, and nausea, vomiting and mucositis were also a problem. All these events occurred in a consolidation combination chemotherapy trial (Cocconi et al, 1999). Especially, in our opinion, patients of maintenance trials were linked to long-lasting intravenous therapy, while our sequential regimen was stopped after 5–6 months.

Further discussion of the advantages and disadvantages of sequential FEC-paclitaxel, of their combination and of induction–maintenance regimens could clearly include evaluations of quality of life (QoL), which were not carried out in this study. We attempted to make some assessment of this aspect in a previous study (Riccardi et al, 2000), but the data were controversial and difficult to evaluate. The poor clinical feasibility of evaluating QoL during and after treatment with the currently used questionnaires is strongly indicated by the very low number of studies in which this has been carried out, especially in MBC (Batel-Copel et al, 1997). Efforts are being made to optimize the use of QoL questionnaires because choosing different techniques leads to different conclusions (Curran et al, 2000).

From this study it appears that sequential first-line FEC-paclitaxel treatment offers the patients a reasonable opportunity of a good antitumour effect while avoiding at least a number of untoward side effects, especially haematologic. This must be taken into account in the overall palliative treatment of MBC, since avoiding side effects of treatment is a means of preserving QoL.

### ACKNOWLEDGEMENTS

Research supported by CNR (Consiglio Nazionale delle Ricerche, Roma, Progetto Finalizzato Applicazioni Cliniche della Ricerca Oncologica, grant. no. 96.00626.PF39), by IRCCS (Istituto di Ricovero e Cura a Carattere Scientifico Policlinico San Matteo, Pavia) and by MURST (Ministero dell' Università e della Ricerca Scientifica e Tecnologica, Roma, Cofinanziamento 1999).

### REFERENCES

Batel-Copel LM, Kornblith AB, Batel PC and Holland JC (1997) Do oncologists have an increasing interest in the quality of life of their patients? A literature review of the last 15 years. *Eur J Cancer* **33**: 29–32

Biganzoli L and Piccart MJ (1997) The bigger the better?...or what we know and what we still need to learn about anthracycline dose per course, dose density and cumulative dose in the treatment of breast cancer. *Ann Oncol* **8**: 1177–1182

Bishop JF, Dewar J, Toner GC, Smith J, Tattersall MH, Olver IN, Ackland S, Kennedy I, Goldstein D, Gurney H, Walpole E, Levi J, Stephenson J and Canetta R (1999) Initial paclitaxel improves outcome compared with CMFP combination chemotherapy as front-line therapy in untreated metastatic breast cancer. *J Clin Oncol* **17**: 2355–2364

Cocconi G, Bisagni G, Bella M, Acito L, Anastasi P, Carpi A, Di Costanzo F, Frassoldati A, Mosconi A, Borrini A and Buzzi P (1999) Comparison of CMF (cyclophosphamide, methotrexate, and 5-fluorouracil) with a rotational crossing and a sequential intensification regimen in advanced breast cancer: a prospective randomized study. *Am J Clin Oncol* **22**: 593–600

Coombes RC, Bliss JM, Wils J, Morvan F, Espie M, Amadori D, Gambrosier P, Richards M, Aapro M, Villar-Grimalt A, McArdle C, Perez-Lopez FR, Vassilopoulos P, Ferreira EP, Chilvers CE, Coombes G, Woods EM and Marty M (1996) Adjuvant cyclophosphamide, methotrexate, and fluorouracil versus fluorouracil, epirubicin, and cyclophosphamide chemotherapy in premenopausal women with axillary node-positive operable breast cancer: results of a randomized trial. *J Clin Oncol* **14**: 35–45

Curran D, Aaronson N, Standaert B, Molenberghs G, Therasse P, Ramirez A, Koopmanschap M, Erder H and Piccart M (2000) Summary measures and statistics in the analysis of quality of life data: an example from an EORTC-NCIC-SAKK locally advanced breast cancer study. *Eur J Cancer* **36**: 834–844

Falkson G, Gelman RS, Pandya KJ, Osborne K, Tormey D, Cummings F, Sledge GW and Abeloff MD (1998) Eastern Cooperative Oncology Group randomized trial of observation versus maintenance therapy for patients with metastatic breast cancer in complete remission following induction treatment. *J Clin Oncol* **16**: 1669–1676

Fossati R, Confalonieri C, Torri V, Ghislandi E, Penna A, Pistotti V, Tinazzi A and Liberati A (1998) Cytotoxic and hormonal treatment for metastatic breast cancer: a systematic review of published randomized trials involving 31,510 women. *J Clin Oncol* **16**: 3439–3460

Gehl J, Boesgaard M, Paaske T, Jensen BV and Dombernowsky P (1996) Paclitaxel and doxorubicin in metastatic breast cancer. *Semin Oncol* **23** (Suppl 15): 35–38

Holmes FA, Walters RS, Theriault RL, Forman AD, Newton LK, Raber MN, Buzdar AU, Frye DK and Hortobagyi GN (1991) Phase II trial of taxol, an active drug in the treatment of metastatic breast cancer. *J Natl Cancer Inst* **83**: 1797–1805

Levine MN, Bramwell VH, Pritchard KI, Norris BD, Shepherd LE, Abu-Zahra H, Findlay B, Warr D, Bowman D, Myles J, Arnold A, Vandenberg T, MacKenzie R, Robert J, Ottaway J, Burnell M, Williams CK and Tu D (1998) Randomized trial of intensive cyclophosphamide, epirubicin, and fluorouracil chemotherapy compared with cyclophosphamide, methotrexate, and fluorouracil in premenopausal women with node-positive breast cancer. National Cancer Institute of Canada Clinical Trials Group. *J Clin Oncol* **16**: 2651–2658

Muss HB, Case LD, Richards F, White DR, Cooper MR, Cruz JM, Powell BL, Spurr CL, Capizzi RL and the Piedmont Oncology Association (1991) Interrupted

*© 2001 Cancer Research Campaign* 

versus continuous chemotherapy in patients with metastatic breast cancer. *NEJM* **325**: 1342–1348

Nabholtz JM, Gelmon K, Bontenbal M, Spielmann M, Catimel G, Conte P, Klaassen U, Namer M, Bonneterre J, Fumoleau P and Winograd B (1996) Multicenter, randomized comparative study of two doses of paclitaxel in patients with metastatic breast cancer. *J Clin Oncol* **14**: 1858–1867

Nabholtz JM, Senn HJ, Bezwoda WR, Melnychuk D, Deschenes L, Douma J, Vandenberg TA, Rapoport B, Rosso R, Trillet-Lenoir V, Drbal J, Molino A, Nortier JW, Richel DJ, Nagykalnai T, Siedlecki P, Wilking N, Genot JY, Hupperets PS, Pannuti F, Skarlos D, Tomiak EM, Murawsky M, Alakl M and Aapro M, for the 304 Study Group (1999) Prospective randomized trial of docetaxel versus mitomycin plus vinblastine in patients with metastatic breast cancer progressing despite previous anthracycline-containing chemotherapy. *J Clin Oncol* **17**: 1413–1424

Pazos C, Mickiewicz E, Di Notto MR, Coppola F, Ventriglia M, Jovtis S, Balbiani L, Lewi D, Rondinon M, Temperley G, Trigo M, Bertoncin AM, Pascual M, Uranga G, Cazap E, Breier S, Grasso S, Estevez R, Triguboff E, Alvarez A and Suarez A (1999) Phase II of doxorubicin/taxol in metastatic breast cancer. *Breast Cancer Res Treat* **55**: 91–96

Pivot X, Asmar L and Hortobagy GN (1999) The efficacy of chemotherapy with docetaxel and paclitaxel in anthracyclin-resistant breast cancer. *Int J Oncol* **15**: 381–386

Pouillart P, Fumoleau P and Romieu G (1999) Final results of a phase II randomized, parallel study of doxorubicin/cycclophosphamide and doxorubicin/Taxol (paclitaxel) as neoadjuvant treatment of local-regional breast cancer [abstract]. *Proc Am Soc Clin Oncol* **18**: 73a

Rahman ZU, Frye DK, Smith TL, Asmar L, Theriault RL, Buzdar AU and Hortobagyi GN (1999) Results and long term follow-up for 1581 patients with metastatic breast carcinoma treated with standard dose doxorubicin-containing chemotherapy. *Cancer* **85**: 104–111

Reichman BS, Seidman AD, Crown JP, Heelan R, Hakes TB, Lebwohl DE, Gilewski TA, Surbone A, Currie V and Hudis CA (1993) Paclitaxel and recombinant human granulocyte colony-stimulating factor as initial chemotherapy for metastatic breast cancer. *J Clin Oncol* **11**: 1943–1951

Riccardi A, Tinelli C, Pugliese P, Brugnatelli S, Giordano M, Danova M, Richetti A, Fava S, Nastasi P, Rinaldi E, Fregoni V, De Monte A and Trotti G (for the Cooperative Group of Study and Treatment of Breast Cancer) (2000) Doubling the epirubicin dosage in the 5-fluorouracil, epirubicin and cyclophosphamide (FEC) regimen for advanced breast cancer: a prospective, randomized, multicentric study on antitumor effect and life quality. *Int J Oncol* **16**: 769–776

Rischin D, Smith J, Millward M, Lewis C, Boyer M, Richardson G, Toner G, Gurney H and McKendrick J (2000) A phase II trial of paclitaxel and epirubicin in advanced breast cancer. *Br J Cancer* **83**: 438–442

Rivera E, Holmes FA, Frye D, Valero V, Theriault RL, Booser D, Walters R, Buzdar AU, Dhingra K, Fraschini G and Hortobagyi GN (2000) Phase II study of paclitaxel in patients with metastatic breast carcinoma refractory to standard chemotherapy. *Cancer* **89**: 2195–2201

Sledge GWJ, Neuberg D, Ingle J, Martino S and Wood W (1997) Phase III trial of doxorubicin (A) vs paclitaxel (T) vs doxorubicin+paclitaxel (A + T) as first line therapy for metastatic breast cancer (MBC): an intergroup trial). *Proc Am Soc Clin Oncol* **16**: abstr. 2

Sparano JA, Hu P, Rao RM, Falkson CI, Wolff AC and Wood WC (1999) Phase II trial of doxorubicin and paclitaxel plus granulocyte colony-stimulating factor in metastatic breast cancer: an Eastern Cooperative Oncology Group Study. *J Clin Oncol* **17**: 3828–3834

Venturini M, Lunardi G, Del Mastro L, Vannozzi MO, Tolino G, Numico G, Viale M, Pastrone I, Angiolini C, Bertelli G, Straneo M, Rosso R and Esposito M (2000) Sequence effect of epirubicin and paclitaxel treatment on pharmacokinetics and toxicity. *J Clin Oncol* **18**: 2116–2125

Winer EP, Morrow M, Osborne CK and Harris JR (2001) Malignant tumors of the breast. In *Cancer Principles and practice of Oncology (6th Ed)*, De Vita VT, Hellman S, Rosenberg SA. (ed) pp 1651–1716. Lippincott Williams and Wilkins, Philadelphia.

 *© 2001 Cancer Research Campaign*
